# Resistance Exercise as a Dual-Target Strategy in Physical Therapy: Reducing Depressive Symptoms to Improve Rehabilitation Participation and Outcomes

**DOI:** 10.1016/j.arrct.2026.100615

**Published:** 2026-03-21

**Authors:** Volodymyr Maksym

**Affiliations:** Department of Physical Rehabilitation and Sports Medicine, Lviv State University of Physical Culture named after Ivan Boberskyi, Lviv, Ukraine

**Keywords:** Adherence, Depressive symptoms, Physical therapy, Rehabilitation, Resistance training, Self-efficacy

## Abstract

Depressive symptoms are prevalent in physical therapy and rehabilitation populations and can act as a limiting factor for rehabilitation participation and outcomes across diagnoses. Even when not meeting criteria for major depressive disorder, elevated depressive symptoms can undermine rehabilitation participation through reduced motivation, impaired self-regulation, sleep disturbance, fatigue, low perceived control, and avoidance of effort or movement. Although referral pathways for mental health care remain essential, access barriers and stigma commonly delay help-seeking. This Special Communication argues that resistance exercise can be positioned in physical therapy as a dual-target intervention: supporting functional recovery while concurrently engaging mood-relevant mediators that influence rehabilitation participation and outcomes. Beyond physiological adaptations, resistance training provides rapid and measurable experiences of mastery and agency (eg, progressive increases in repetitions, time under tension, or load), which may strengthen self-efficacy and perceived control, reduce helplessness, and improve tolerance to discomfort through graded exposure to effort. These mediators are clinically relevant because they map directly onto modifiable drivers of adherence, persistence, and dropout risk in rehabilitation. Pragmatic, safety-oriented principles are outlined for prescribing resistance exercise across settings (clinic- or home-based; minimal equipment), including techniques to maximize early achievable success, simplify programs to reduce cognitive load, apply symptom- and fatigue-guided progression, and monitor engagement outcomes. Scope of practice boundaries and red flags requiring timely referral are also discussed. Future work should test implementation strategies and quantify whether targeting mood-related mediators through resistance exercise improves both depressive symptoms and rehabilitation endpoints, including adherence, functional outcomes, and return to valued activities.

Depressive symptoms are common among people seeking physical therapy and rehabilitation. They may predate the index condition, emerge in response to pain, disability, and life disruption, or fluctuate with the medical course and social stressors. Regardless of etiology, elevated depressive symptoms can materially influence rehabilitation processes, including reduced attendance, lower engagement during sessions, inconsistent home program adherence, and a higher risk of dropout.^1^, ^2^, ^3^, ^4^, ^5^, ^6^ These behavioral consequences are clinically important because they shape exposure to therapeutic dose—how often a patient attends, how much they do, and whether progression occurs—thereby influencing functional recovery. In this context, depressive symptoms should be viewed not only as a comorbidity but also as a modifiable barrier to rehabilitation dose and outcomes. Systematic reviews in musculoskeletal physiotherapy identify depression, low self-efficacy, helplessness, and perceived barriers as consistent predictors of poor treatment adherence.^5^

Rehabilitation professionals appropriately emphasize differential diagnosis, tissue healing, symptom modulation, and graded functional restoration. Yet when rehabilitation “stalls” despite technically sound programming, contributors are sometimes framed as motivation problems or “poor compliance.” Such framing can obscure modifiable mechanisms that frequently accompany depressive symptoms: fatigue, sleep disruption, low perceived control, helplessness, anticipatory avoidance, and cognitive-affective amplification of symptoms.^6^, ^7^, ^8^ These factors can be conceptualized as mood-related mediators that sit between depressive symptoms and rehabilitation outcomes.

Mental health referral pathways remain essential, particularly for severe presentations and high-risk situations. However, access barriers and stigma commonly delay help-seeking.^9^^,^^10^ Physical therapists often represent a trusted point of contact and may be among the first clinicians to observe mood-related barriers that limit rehabilitation participation.

## Purpose

This Special Communication proposes resistance exercise as a dual-target strategy in physical therapy to support functional recovery while reducing depressive symptoms and improving rehabilitation participation through modifiable mood-related mediators (eg, self-efficacy/perceived control, mastery experiences, and avoidance).

### Why resistance exercise is uniquely positioned in physical therapy

Exercise is a broad category; not all modalities offer the same psychological and practical affordances in rehabilitation settings. Aerobic exercise is often prescribed for mood, yet it can be limited by pain, breathlessness, intolerance to sustained activity, or contextual barriers (weather, equipment, and safe spaces). In contrast, resistance training (RT) can be delivered in brief, controllable bouts with flexible modification (range of motion, tempo, isometrics, external support, and load), which is often compatible with pain and disability.

A second distinctive property of RT is the immediacy and objectivity of progress feedback. RT offers multiple trackable metrics even when symptoms fluctuate: repetitions, load, time under tension, isometric hold duration, range control, or perceived stability. Such metrics allow the clinician and patient to identify “wins” that are not dependent on pain relief. This matters clinically because pain improvement may be nonlinear in many rehabilitation trajectories. When progress can be demonstrated despite persistent symptoms, patients may be less likely to interpret symptoms as evidence of failure and more likely to remain engaged.

Third, RT can be integrated seamlessly into existing rehabilitation goals. Strength, power, and capacity are central to activities of daily living and return-to-work objectives. A single RT-based exercise can simultaneously target a functional limitation (eg, sit-to-stand strength), a behavioral goal (practice consistency), and a psychological mediator (mastery and control). This alignment supports feasibility: it does not require the therapist to “add a mental health program,” but rather to deliver a core rehabilitation component with explicit attention to its engagement-relevant effects.

Meta-analytic evidence suggests RT is associated with reductions in depressive symptoms across diverse populations, including adults with varying baseline mental health status.^11^, ^12^, ^13^ Although effect sizes vary and studies are heterogeneous, the consistency of direction supports clinical interest. RT’s scalability across settings—clinic, community, or home-based with minimal equipment—makes it a plausible candidate for addressing mood-related barriers within rehabilitation pathways, particularly when psychotherapy or pharmacotherapy access is delayed.

### Mood-related mediators that link depressive symptoms to rehabilitation outcomes

A mediator is clinically meaningful if it helps explain how depressive symptoms translate into reduced rehabilitation participation and poorer outcomes. Several mood-related mediators plausibly link depressive symptoms to rehabilitation outcomes and may be modifiable through resistance exercise design.

### Self-efficacy and perceived control

Self-efficacy—belief in one’s capacity to execute actions required to manage prospective situations—is a robust predictor of effort, persistence, and behavior change.^14^ Depressive symptoms can erode expectancy that effort will lead to improvement (“nothing will help”), reducing willingness to engage with graded challenges. RT can counter this through repeated mastery experiences and objective feedback. From a rehabilitation perspective, self-efficacy is not an abstract construct; it can present as willingness to attempt harder tasks, consistency with home programming, and readiness to progress.

### Helplessness and demoralization

In rehabilitation settings, depressive symptoms may present as reduced initiative, lower expectation that effort will be beneficial, and diminished persistence with rehabilitation tasks.^1^^,^^3^, ^4^, ^5^ Clinically, this may be observed as minimal effort, reluctance to progress, or passive dependence on the clinician.^5^ RT sessions can be designed to emphasize achievable goals and immediate reinforcement (eg, completing a set at tolerable effort, improved form, or increased time under tension). These “safe wins” may help restore agency and support ongoing participation by creating repeated experiences in which effort is followed by a visible, controllable gain.^14^

### Avoidance and threat appraisal

In chronic pain and disability, depressive symptoms frequently overlap with avoidance of activity and heightened threat appraisal, described in fear-avoidance frameworks.^6^, ^7^, ^8^ RT can function as graded exposure to effort: controlled, time-limited bouts of exertion framed as safe and meaningful. Over time, this can improve tolerance to discomfort and reduce avoidance patterns that restrict rehabilitation dose.

### Sleep, fatigue, and self-regulation capacity

Sleep disturbance and fatigue are common depressive symptoms and strongly influence participation capacity. RT may improve sleep quality for some individuals,^15^ potentially supporting rehabilitation participation indirectly. Importantly, fatigue management is also a programming issue: dosing RT to avoid post-exertional “crashes,” and adjusting volume to match recovery capacity, can preserve engagement.

These mediators can be assessed pragmatically through patient-reported measures (eg, sleep quality, fatigue, perceived control) and through behavioral endpoints (attendance, completion of home sessions, willingness to progress). The mediators align with the rehabilitation scope: they influence participation and can be addressed through education, graded exposure, goal setting, and safe progression. This framing is consistent with an ICF-informed rehabilitation perspective in which personal factors (including depressive symptoms) influence activity and participation outcomes alongside body functions and structures.^16^

### Mechanistic plausibility without overclaiming

Positioning RT as a dual-target intervention is most defensible when the proposed mechanisms are plausible and observable in clinical practice, without implying that RT substitutes for evidence-based mental health treatments.

First, RT provides repeated opportunities for mastery experiences, which are central to self-efficacy development.^14^ When the patient observes consistent improvement in a controllable metric (even a small one), the experience can contradict global negative expectations that often accompany depressive symptoms. Second, RT can be structured to reduce avoidance through graded exposure: the patient practices effort and tolerable discomfort in a safe, time-bounded format, which may reduce fear of movement and effort-related avoidance.^5^ Third, RT often supports improvements in functional capacity that are meaningful to daily life. Functional gains can feed back into mood-relevant domains through increased activity participation, social engagement, and perceived competence.

Notably, these mechanisms are compatible with a rehabilitation clinician’s intent: to increase capacity and participation. The “psychological lever” is not a separate psychotherapeutic technique; it is a framing and programming approach that leverages established rehabilitation practice to target mediators that commonly derail adherence.

### RT as a dual-target intervention: practical principles for delivery

This paper does not propose a separate “mental health workout.” Rather, it argues for designing RT within physical therapy to maximize both functional gains and engagement-relevant mediators. The principles below are intended to be feasible, safety-oriented, and applicable across diagnoses.

#### Prioritize early achievable success (“safe wins”)

Early experiences shape expectations. Start with movements the patient can perform successfully with acceptable symptoms and stable technique. Choose exercises that allow rapid scaling (range of motion, tempo, isometrics, external support, or reduced load). Provide explicit feedback linking effort to measurable change (eg, “You held that position longer than last week; this indicates your capacity is changing.”). For patients with low perceived control, visible progress is often more persuasive than reassurance.

#### Use simple patterns and reduce cognitive load

Overly complex programs can increase performance anxiety and reduce adherence. Favor basic movement patterns (hinge, squat-to-chair, step-up, push, pull, and carry) and isometrics when appropriate. Keep cues minimal and consistent. Simplicity supports autonomy and improves the likelihood of accurate home-based execution.

#### Dose effort to be tolerable and repeatable

For engagement goals, maximal or near-maximal RT is rarely necessary. Use symptom-guided intensity (eg, a moderate rating of perceived exertion [RPE] range such as 4-7/10) and avoid programming that reliably triggers prolonged symptom flares or excessive fatigue.^17^ Repeatability supports consistency, which is often a stronger determinant of outcomes than intermittent high-intensity sessions. Mood-related benefits of RT have been reported across a range of intensities, suggesting that near-maximal loading is not universally required for depressive-symptom improvement.^11^, ^12^, ^13^ Autoregulated approaches (eg, RPE- or repetitions-in-reserve–guided progression) may improve feasibility when pain and fatigue fluctuate.^18^ Pain during therapeutic exercise need not be a barrier to successful outcomes in chronic musculoskeletal pain.^19^

#### Progress slowly, but make progress visible

Progression can be a therapeutic tool. Emphasize microprogression: small increases in repetitions, an additional set, a longer isometric hold, modest range expansion, or improved tempo control. Microprogression preserves safety while maintaining a clear signal of change. Visible progress strengthens mastery and perceived control even when pain reduction is slow.

#### Build autonomy through structured choice

Depressive symptoms can reduce initiative; offering limited, structured choices can improve engagement (eg, “Do you prefer band rows or supported dumbbell rows today?”). Autonomy supports ownership and reduces resistance, especially in home-based programs.

#### Integrate goal setting around valued activities

Link RT to patient-valued outcomes rather than abstract strength goals (carrying groceries, climbing stairs, returning to work tasks). Value-based goals can sustain effort when mood is low, and they frame RT as meaningful participation rather than “exercise for exercise’s sake*.*”

#### Monitor fatigue, sleep, and symptom response

Because fatigue and sleep disturbance can drive dropout, monitor these as routine vitals. Adjust volume and intensity accordingly. If a patient consistently reports worsened sleep and marked fatigue after sessions, the program may require lower volume, longer rest intervals, altered exercise selection, or a slower progression rate. Monitoring also reinforces collaboration and control, which supports engagement.

#### Ensure feasibility across settings

A dual-target framing is only useful if implementable. RT can be delivered with bodyweight, bands, or minimal equipment. Home programs should be short, consistent, and trackable (a simple log of sets/reps or hold times). Trackability itself is part of the mechanism: it reinforces mastery and agency.

Table 1 provides an evidence-informed mapping between resistance exercise features, mood-related mediators, and rehabilitation-relevant outcomes, drawing on literature on RT and depressive symptoms, self-efficacy/mastery, fear-avoidance, sleep/fatigue, and adherence.^5^, ^6^, ^7^, ^8^^,^^11^, ^12^, ^13^, ^14^, ^15^, ^16^^,^^17^^,^^20^, ^21^, ^22^, ^23^, ^24^, ^25^, ^26^

**Table 1:** Resistance exercise features, mood-related mediators, and rehabilitation outcomes

RT Feature in PT (Feasible and Scalable)	Mood-Related Mediators Engaged	Rehabilitation-Relevant Outcomes Likely Influenced
Trackable progress (reps, hold time, load, and ROM control)	Increased mastery, self-efficacy, and perceived control	Better adherence, higher session engagement, and reduced dropout
Simple movement patterns and clear cues	Reduced cognitive load and performance anxiety	Greater consistency, better carryover to home programs
Graded exposure to effort (tolerable intensity and stepwise progression)	Reduced helplessness and avoidance; improved coping with discomfort	Higher activity tolerance; improved functional progression
Early “safe wins” (technique-first and achievable doses)	Increased hope/optimism; reduced demoralization	Improved attendance and willingness to progress
Structured autonomy (limited choices; flexible equipment)	Increased autonomy/agency; reduced resistance to referral discussions	Improved long-term maintenance and self-management
Symptom- and fatigue-guided dosing	Improved energy regulation; reduced postexertional setbacks	Fewer setbacks, improved continuity of care
Pain-compatible modifications (isometrics, tempo, and range modulation)	Reduced threat appraisal; reduced fear of movement	Better pain–function balance, improved participation
Therapist feedback and collaborative goal setting	Increased support/relatedness; increased confidence	Stronger therapeutic alliance, improved persistence

NOTE. The table summarizes an evidence-informed conceptual mapping discussed in the manuscript; it is not intended to represent direct causal estimates.

### Minimal dosing examples (illustrative, not prescriptive)

The intent of the examples below is to demonstrate how RT can be delivered with a low threshold for entry, visible progress metrics, and symptom-guided progression. These are not intended as a universal protocol.

### Example A: home-based, minimal equipment (bands + bodyweight)

Two to 3 sessions per week; 20-30 minutes. Select 4-5 exercises: sit-to-stand or supported squat-to-chair; band row; wall or incline push-up; hip hinge pattern (eg, dowel hinge) or bridge; isometric hold (wall sit or plank variant). Begin with 1-2 sets at moderate RPE, prioritizing stable technique. Track one metric per exercise (reps, hold time, or tempo control). Progress by adding 1-2 reps or 5-10 seconds of hold time weekly if symptom response is acceptable.

### Example B: clinic-based (machines or free weights)

Two sessions per week; 30-45 minutes. Use 3-4 primary patterns with safe setup: leg press or sit-to-stand loading; cable row; chest press or supported push; carry or step-up. Begin with conservative loads and clear stopping rules (pain/instability thresholds). Track performance in a simple log and emphasize microprogression. If fatigue is prominent, reduce total volume (fewer sets) before reducing frequency, to preserve routine and continuity.

### Example C: pain-sensitive or flare-prone presentations (isometrics and tempo)

Two to 3 sessions per week; 15-25 minutes. Use isometrics (eg, mid-range holds) and slow tempo at short ranges to allow tolerable loading. Track hold time or perceived steadiness rather than load. Progress primarily through hold time and range expansion, then add external resistance as tolerated.

### Implementation in real-world rehabilitation settings

For a dual-target approach to be clinically useful, it must fit workflow constraints. Several practical steps can increase feasibility without adding substantial burden.

Use routine language that normalizes mood-related barriers. Simple, neutral statements can reduce stigma and resistance: “We track mood and sleep because they affect recovery, just like pain and fatigue.” This framing positions mood-related factors as part of rehabilitation science rather than a character judgment.

Measure engagement outcomes explicitly. If RT is intended to improve adherence and persistence, those endpoints should be observed. Attendance, completion of home sessions, and willingness to progress are clinically meaningful outcomes that can be tracked with minimal effort (eg, a weekly check-in question plus an exercise log). These metrics can guide clinical decision-making and support shared problem-solving. Systematic reviews of physiotherapy adherence highlight the value of monitoring, feedback, and motivational or cognitive-behavioral strategies to improve attendance and adherence.^5^^,^^21^^,^^22^

Design for “minimum effective complexity.” Many adherence failures occur because programs are too complex or too time-intensive. A brief, repeatable RT routine with clear tracking can be more effective than an ambitious program completed sporadically. Where possible, consolidate exercise selection to a small set of patterns and retain them long enough for the patient to experience mastery. Behavior change techniques, such as goal setting, self-monitoring, and feedback, have been examined in physiotherapy and physical-activity adherence interventions and can support engagement.^23^, ^24^, ^25^, ^26^

Plan for inevitable variability. Mood, pain, and fatigue fluctuate. A dual-target approach anticipates variability and includes permission to adjust intensity and volume while preserving routine. This reduces all-or-nothing thinking (“I missed a session so I failed”) and supports continuity.

In frail or medically complex patients (eg, postacute rehabilitation, hemiplegia, cognitive impairment, or end-of-day fatigue in inpatient settings), the proposed approach may require shorter bouts, fewer exercises, and a stronger emphasis on assisted setup, supervised technique, and conservative progression. In these contexts, the “minimum effective complexity” principle becomes particularly important to preserve participation without exacerbating fatigue or symptoms. Similar programs and personal factors (including cognitive status and mood) influence adherence in older adults.^20^

### Safety, scope of practice, and referral boundaries

Framing RT as a dual-target intervention does not imply that physical therapists diagnose or treat depressive disorders; rather, they screen and monitor symptoms and use established referral pathways when severe or high-risk presentations are suspected. Physical therapists can appropriately (1) recognize mood-related barriers affecting participation, (2) screen or monitor symptoms using validated tools consistent with local scope and training, and (3) maintain clear referral pathways.^27^^,^^28^

Red flags and urgent referral. High-risk indicators require immediate action consistent with institutional policy and local regulations, including disclosure of active self-harm intent, severe functional deterioration with marked psychomotor slowing, or symptoms suggesting psychosis or mania. If a patient endorses thoughts of self-harm on a screening item (eg, item 9 of the Patient Health Questionnaire-9), clinicians should follow a predefined safety protocol, involve appropriate medical/mental health services, and not rely on exercise as the primary response.

Adverse events and contraindications. RT should be prescribed consistently with standard rehabilitation safety: medical screening as indicated, attention to cardiovascular risk, post-surgical precautions, neurologic considerations, and symptom-guided progression.^17^ In this model, minimizing early adverse experiences is also psychologically protective; repeated flares can reinforce helplessness and avoidance. Delayed onset muscle soreness, particularly after novel eccentric loading, may transiently increase pain and reduce adherence; early programming can limit excessive eccentric volume and progress gradually.^29^

### Study limitations and guardrails

Several limitations warrant explicit acknowledgment. First, evidence for RT reducing depressive symptoms is heterogeneous; outcomes depend on population, baseline symptom severity, co-interventions, adherence, and study design.^11^, ^12^, ^13^ Second, depressive symptoms may reflect broader social stressors and clinical comorbidities that are not modifiable through exercise alone. RT should be viewed as one accessible component within stepped, multidisciplinary care rather than a replacement for psychotherapy or pharmacotherapy when indicated. Third, there is a risk of overattribution: improvement in mood-related mediators may coincide with natural recovery or other rehabilitation components. Pragmatic trials and mediation analyses are needed to test whether changes in proposed mediators explain downstream changes in rehabilitation outcomes.

Finally, implementation requires clinician comfort and clear boundaries. Therapists should avoid making diagnostic statements and should use structured referral pathways when severe symptoms or risk indicators are present. The dual-target framing is most defensible when presented as “supporting participation and recovery” while coordinating care with appropriate mental health services.

### Implications for research and implementation

To evaluate RT as a dual-target intervention in physical therapy, research should move beyond symptom scores alone. Depression measures remain important, but rehabilitation endpoints such as adherence, engagement, attendance, dropout, time-to-goal, functional outcomes, and return to valued activity may be equally informative. Pragmatic trials and implementation studies are particularly relevant because the proposed approach is designed for real-world constraints.

Key research questions include:

(1) Which patients benefit most (baseline depressive symptoms, pain phenotypes, disability severity)?
(2) Which RT features best engage specific mediators (mastery vs sleep/energy vs avoidance)?
(3) What is the minimal effective dose and progression strategy that preserves feasibility and safety in rehabilitation populations?
(4) Do improvements in mediators (self-efficacy, perceived control, reduced avoidance) explain downstream changes in rehabilitation outcomes?

## Conclusions

Depressive symptoms can act as a powerful, often under-recognized constraint on rehabilitation participation and outcomes. Resistance exercise, already foundational within physical therapy, can be intentionally framed and delivered as a dual-target intervention: advancing functional recovery while engaging mood-related mediators that influence adherence and persistence. By emphasizing achievable early success, trackable progress, symptom-guided dosing, and autonomy-supportive programming, clinicians may improve both engagement and outcomes for patients whose recovery is limited by mood-related barriers. Clear scope boundaries and referral pathways remain essential, particularly for severe or high-risk presentations. Future pragmatic research should test whether targeting mood-related mediators through resistance exercise improves not only depressive symptoms but also rehabilitation engagement, functional outcomes, and sustained participation in valued life activities.
